# Supplementary material for: Visualizing multiple inter-organelle contact sites using the organelle-targeted split-GFP system
Source: Sci Rep. 2018 Apr 18;8:6175. doi: 10.1038/s41598-018-24466-0 (PMC5906596; doi:10.1038/s41598-018-24466-0)
Supplement: Supplementary file 1 — SUPPLEMENTARY INFORMATION [file 41598_2018_24466_MOESM1_ESM.pdf]

Supplementary Information

Visualizing multiple inter-organelle contact sites using the organelle-targeted split-GFP system

Yuriko Kakimoto[1], Shinya Tashiro[1], Rieko Kojima[1], Yuki Morozumi[1], Toshiya Endo[2] & Yasushi Tamura[1]*

[1] Faculty of Science, Yamagata University, 1-4-12 Kojirakawa-machi, Yamagata, Yamagata 990-8560, Japan. [2] Faculty of Life Sciences, Kyoto Sangyo University, Kamigamo-motoyama, Kita-ku, Kyoto 603-8555, Japan.

*Correspondence and requests for materials should be addressed to Y.T. (email:tamura@sci.kj.yamagata-u.ac.jp)

Contents

Supplementary figures S1 to S6, Materials and Table S1 and S2

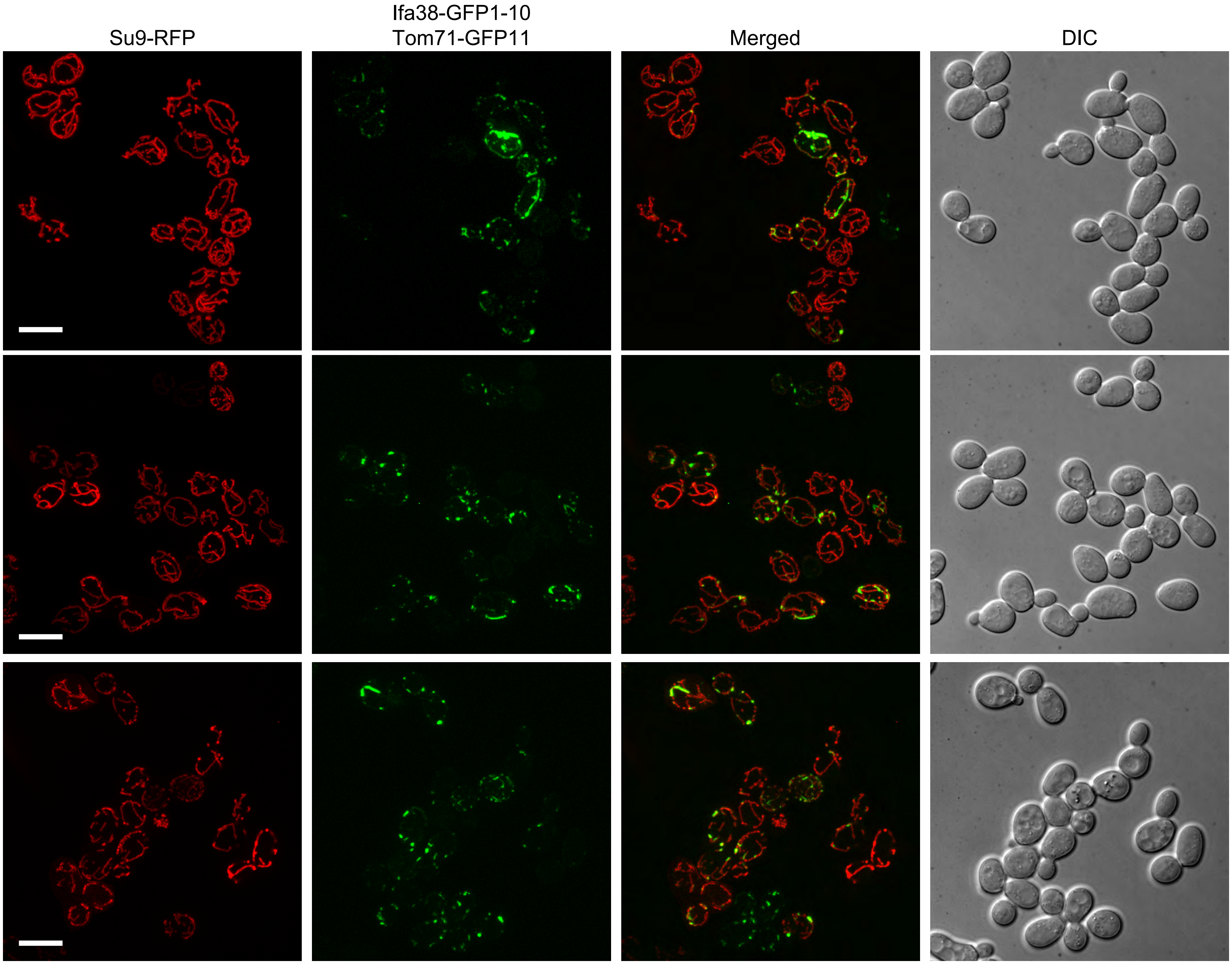

Kakimoto et al., Figure S1

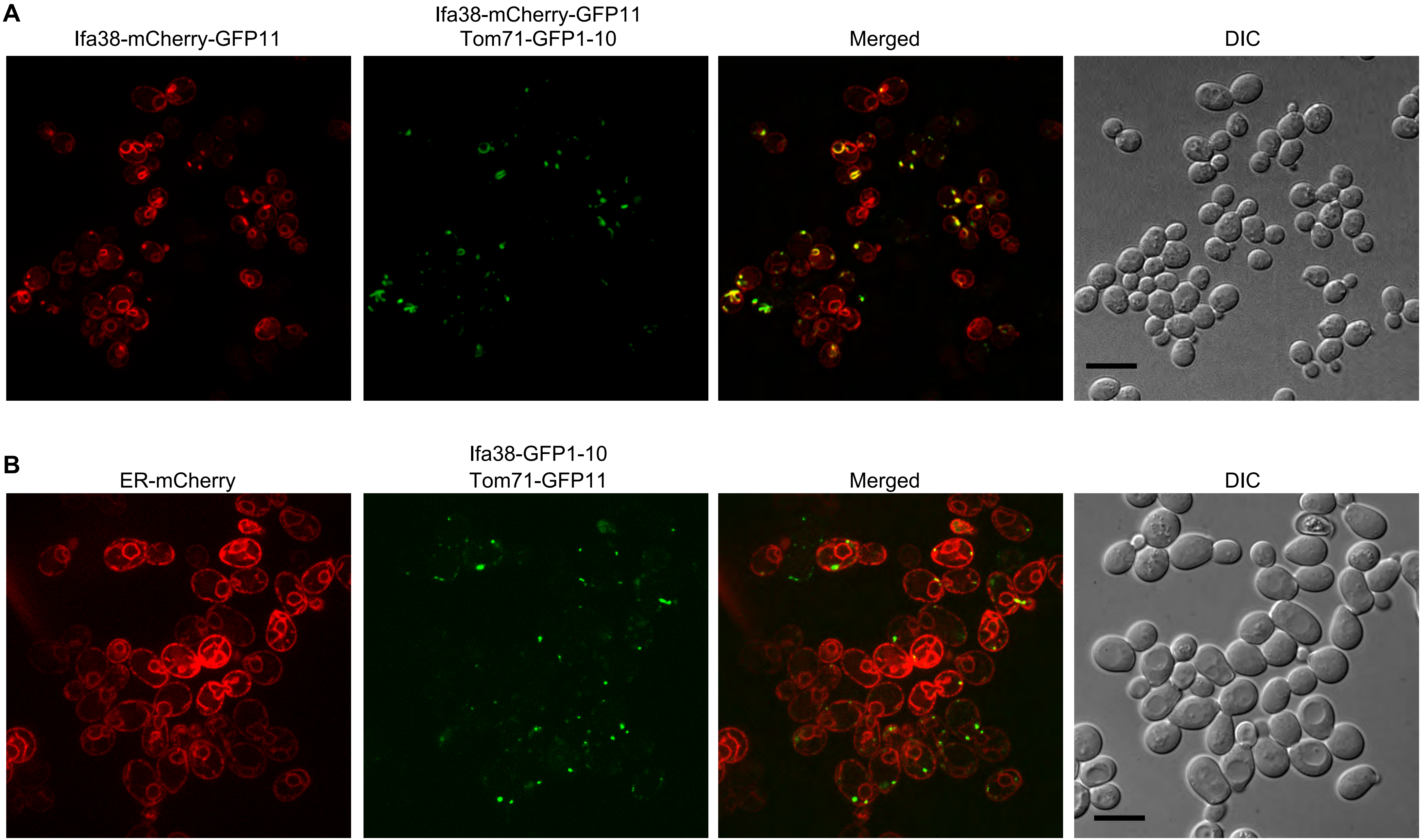

Kakimoto et al., Figure S2

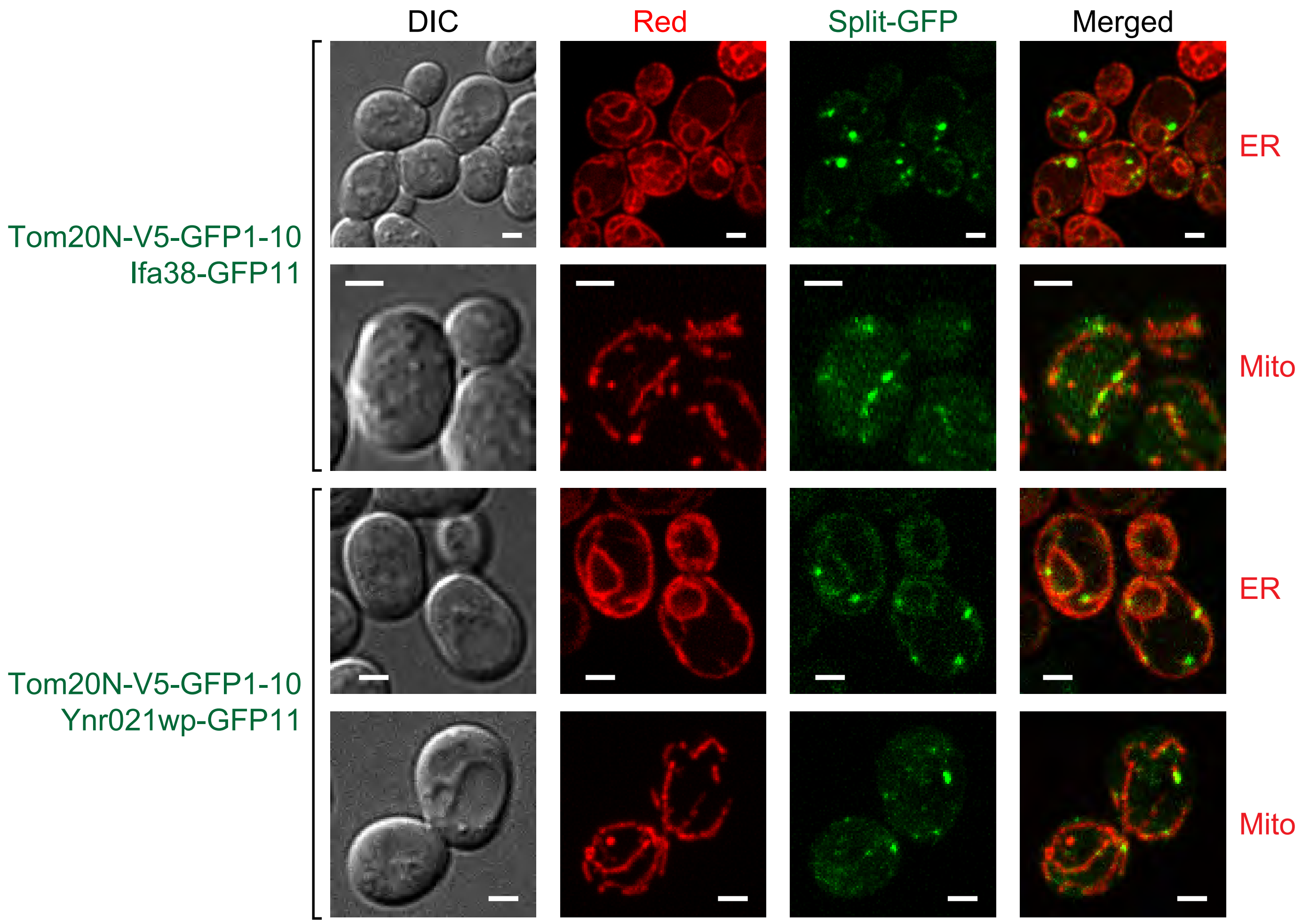

Kakimoto et al., Figure S3

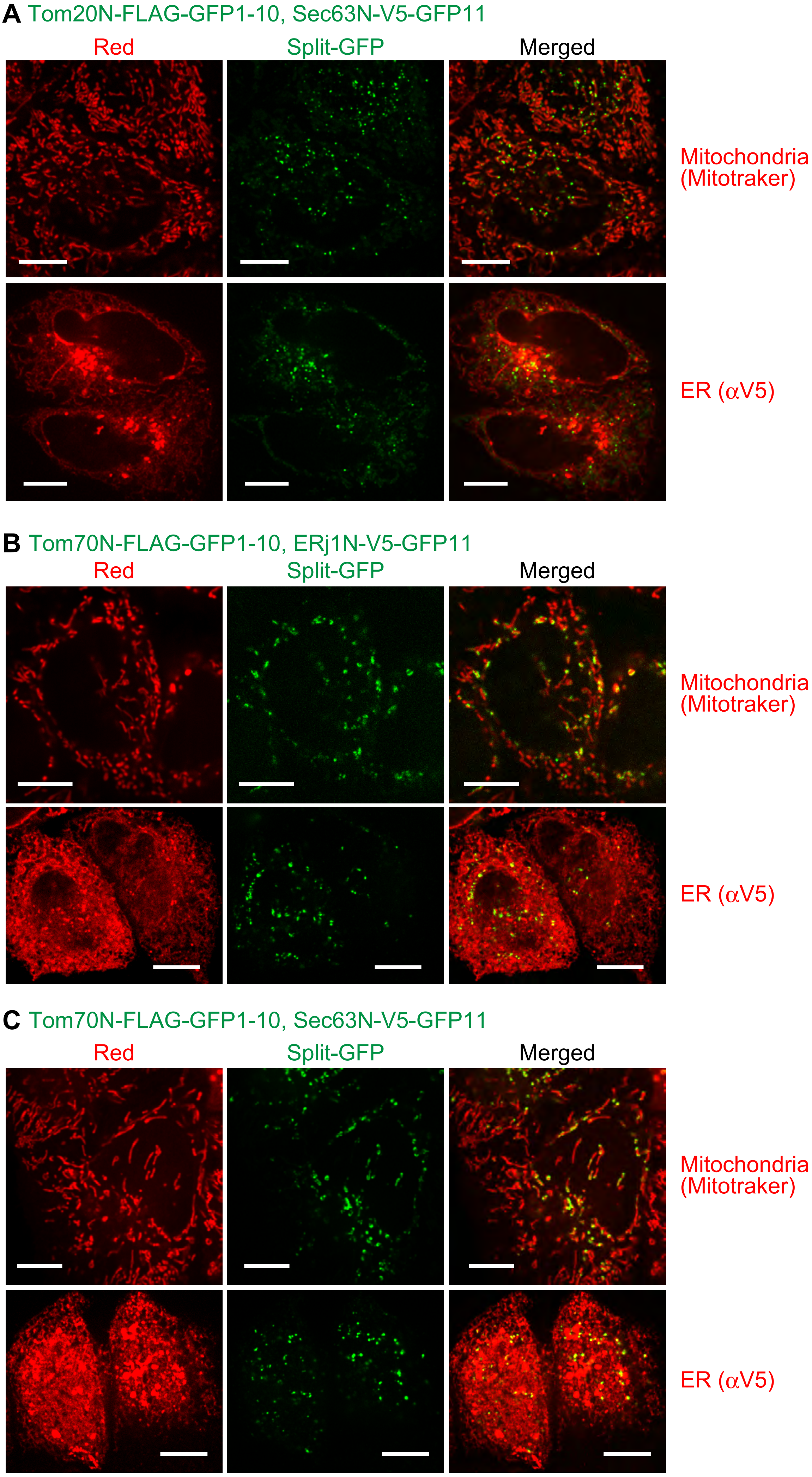

Kakimoto et al., Figure S4

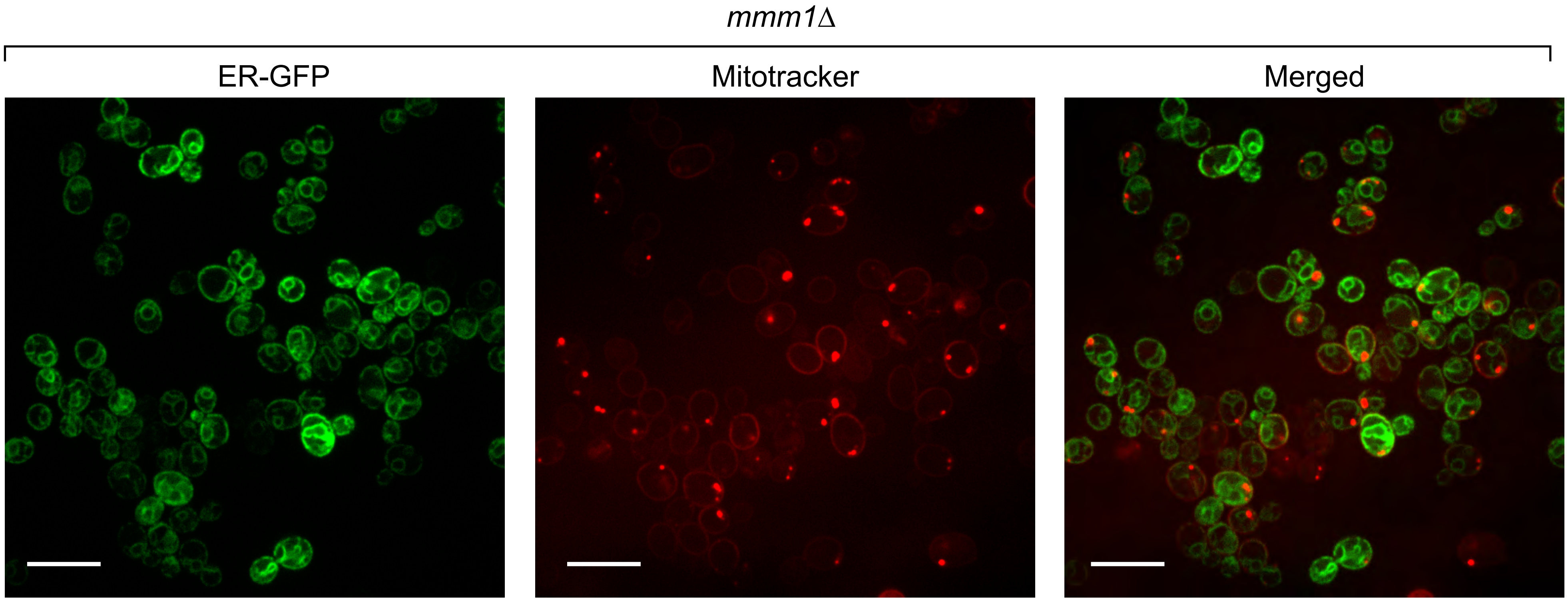

Kakimoto et al., Figure S5

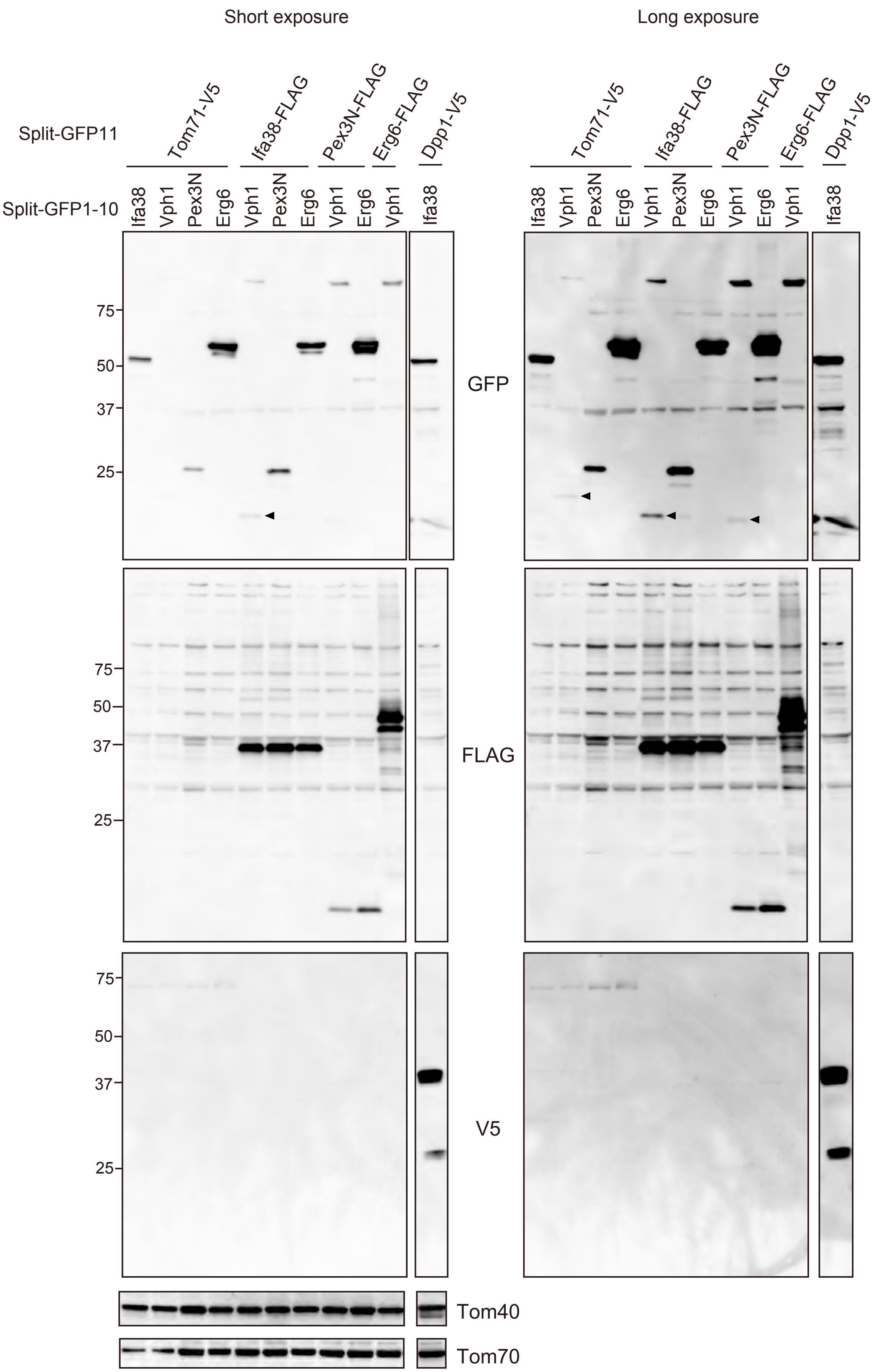

Kakimoto et al., Figure S6

Figures S1 and S2. Representative images showing cells expressing split-GFP probes for visualizing the ER-mitochondria contact sites. Yeast cells expressing Ifa38-GFP1-10 and Tom71- GFP11 (S1), Ifa38-mCherry-GFP and Tom71-GFP1-10 (S2A) and Ifa38-GFP1-10 and Tom71-GFP11 (S2B) were imaged by confocal fluorescent microscopy. Maximum projection images of whole visual microscope field were reconstituted from the z-stacks and were shown. Scale bar represents 10 μm.

Figure S3. The ER-mitochondria contact sites visualized with split-GFP fragments in yeast.

Yeast cells expressing split-GFP proteins, Tom20N-V5-GFP1-10 and Ifa38-GFP11 or Ynr021wp-GFP11, and mitochondria-targeted RFP (Mito) or the ER-targeted mCherry (ER) were imaged by confocal fluorescent microscopy. Scale bar represents 2 μm.

Figure S4. The ER-mitochondria contact sites visualized with the split-GFP system in HeLa cells.

A-C HeLa cells transiently expressing Tom20N-FLAG-GFP1-10 and Sec63N-V5-GFP11 (A), Tom70N-FLAG-GFP1-10 and ERj1N-V5-GFP11 (B), Tom70N-FLAG-GFP1-10 and Sec63N-V5-GFP11 (C), were stained with MitoTracker and immediately observed (upper panel) or fixed and subjected to immunofluorescence using anti-V5 antibodies (lower panel). Scale bar represents 10 μm.

Figure S5. The ER and mitochondria associate in the absence of Mmm1. Yeast cells expressing ER-targeted GFP were stained with MitoTracker Red CMXRos and imaged by confocal fluorescent microscopy. Single focal plane images of whole visual microscope field were shown. Scale bar represents 10 μm.

Figure S6. Immunoblotting of total cell lysates showing the expression levels of split-GFP probes. Total cell lysates were prepared from logarithmically growing cells expressing the indicated split-GFP fusion proteins and were analyzed by immunoblotting using the indicated antibodies. Arrowheads indicate degraded products

from Vph1-GFP1-10.

Materials

DNA sequences for the split-GFP fragments and organelle-targeting signals

GFP1-10

CATGGGTGGCACTAGTATGAGCAAAGGAGAAGAACTTTTCACTGGAGTTGT
CCCAATTCTTGTTGAATTAGATGGTGATGTTAATGGGCACAAATTTTCTGTC
AGAGGAGAGGGTGAAGGTGATGCTACAATCGGAAAACTCACCCTTAAATTT
ATTTGCACTACTGGAAAACTACCTGTTCCATGGCCAACACTTGTCACTACTC
TGACCTATGGTGTTCAATGCTTTTCCCGTTATCCGGATCACATGAAAAGGCA
TGACTTTTTCAAGAGTGCCATGCCCGAAGGTTATGTACAGGAACGCACTATA
TCTTTCAAAGATGACGGGAAATACAAGACGCGTGCTGTAGTCAAGTTTGAA
GGTGATACCCTTGTTAATCGTATCGAGTTAAAGGGTACTGATTTTAAAGAAG
ATGGAAACATTCTCGGACACAAACTCGAGTACAACTTTAACTCACACAATG
TATACATCACGGCAGACAAACAAAAGAATGGAATCAAAGCTAACTTCACAG
TTCGCCACAACGTTGAAGATGGTTCCGTTCAACTAGCAGACCATTATCAACA
AAATACTCCAATTGGCGATGGCCCTGTCCTTTTACCAGACAACCATTACCTG
TCGACACAAACTGTCCTTTCGAAAGATCCCAACGAAAAGTAA

GFP11

CGATGGAGGGTCTGGTGGCGGATCAACAAGTCGTGACCACATGGTCCTTCA
TGAGTACGTAAATGCTGCTGGGATTACATAA

3xFLAG-GFP11

TCTGCCGGTGGTGATTACAAAGACCATGATGGCGATTATAAGGATCATGAC
ATTGACTATAAGGATGATGACGATAAAGGCGGAAGTGGTGGAGGTTCAACT
TCCAGAGATCACATGGTTTTGCATGAATACGTCAATGCTGCAGGGATAACA
TAA

V5-GFP11

TCTGCCGGAGGTTCTGGGAAACCCATACCAAACCCTCTTTTGGGCTTAGACT
CAACTGGTGGAAGTGGTGGTGGTTCCACAAGCAGAGATCATATGGTTCTAC
ACGAATATGTCAATGCTGCAGGCATTACCTAA

ERj1(1-200)-V5

GCCACCATGACTGCTCCGTGCTCTCAACCCGCACAACTGCCAGGACGGAGA
CAGCTGGGTCTGGTACCTTTTCCTCCTCCACCACCACGTACACCTCTGCTGT
GGTTGCTGCTTCTGCTGCTTGCTGCGGTTGCACCGGCTAGAGGATGGGAATC
CGGCGATTTGGAGCTGTTTGACCTGGTCGAAGAAGTGCAGTTGAACTTCTAC
CAGTTTCTCGGGGTACAACAGGATGCCAGCTCAGCTGATATCCGCAAAGCC
TATCGGAAATTGTCCCTCACACTGCATCCCGATAAGAACAAAGACGAGAAT
GCCGAAACCCAGTTCAGACAGCTCGTGGCCATTTACGAGGTCCTGAAAGAC
GATGAGAGGAGACAGAGGTATGACGACATTCTGATCAACGGACTGCCCGAC
TGGCGACAGCCTGTGTTCTACTATAGGCGAGTGCGGAAGATGTCCAATGCC
GAGCTTGCCCTGCTTCTCTTCATCATTCTCACGGTTGGCCACTATGCAGTCGT
GTGGAGCATCTACCTGGAGAAACAGCTGGATGAGCTCCTGTCACGCAAGAA
GCGCGAAAAGAAGAAGAAAACCGGCAGTAAGAGCGTGGATGGGAAACCCA
TACCCAATCCACTCCTGGGTCTTGACTCTACTGGAGGGAGTGGCGGC

Sec63(1-240)-V5

GCCACCATGGCCGGACAACAGTTCCAGTATGACGACTCAGGGAACACCTTC
TTCTACTTCCTGACATCCTTCGTCGGCCTGATCGTCATACCAGCGACCTACT
ACCTCTGGCCCAGAGATCAGAACGCAGAACAGATACGGCTCAAGAACATTC
GTAAGGTGTATGGACGGTGCATGTGGTACCGCCTTAGACTGCTGAAACCTC
AACCCAACATCATACCCACTGTCAAGAAAATCGTGCTGTTGGCAGGATGGG
CTCTGTTTCTCTTTCTGGCCTACAAAGTGAGCAAGACAGACAGGGAGTATCA
GGAGTACAACCCGTATGAAGTGCTCAATCTTGATCCAGGCGCTACAGTGGC
CGAAATCAAGAAGCAGTATCGATTGTTGAGCCTCAAGTATCACCCCGATAA
AGGAGGCGATGAGGTGATGTTTATGCGCATTGCCAAAGCGTATGCAGCACT
GACTGACGAAGAGTCTAGGAAGAATTGGGAGGAGTTTGGGAATCCTGATGG
TCCACAAGCTACCTCCTTTGGTATCGCCCTTCCTGCTTGGATCGTTGACCAG
AAGAACAGTATTCTGGTCCTGCTGGTTTACGGACTTGCCTTTATGGTGATTC

TGCCAGTAGTAGTTGGGTCATGGTGGTATAGGTCCATCAGATACAGCGGGG
ACCAGATTCTGATTCGGACGACACAGATTTACACCTACTTCGTGTACAAAAC
CCGCAATGGCAAACCTATCCCGAATCCCCTGTTGGGGCTGGATAGTACTGG
CGGTTCTGGCGGC

Tom20(1-33)-FLAG

GCCACCATGGTGGGCAGGAACAGCGCCATCGCCGCCGGCGTGTGCGGCGCC
CTGTTCATCGGCTACTGTATCTATTTTGACCGGAAGCGGAGATCCGACCCCA
ATGATTACAAGGACCACGATGGCGACTATAAGGATCACGACATCGATTACA
AGGACGATGACGATAAGGGCGGCTCTGGCG

Tom70(1-70)-3XFLAG

GCCACCATGGCCGCGTCCAAACCCGTGGAAGCAGCCGTCGTTGCAGCCGCT
GTGCCGAGCTCTGGGTCAGGCGTGGGTGGAGGAGGAACCGCTGGTCCCGGA
ACAGGCGGCCTTCCTCGGTGGCAGTTGGCCCTGGCTGTAGGGGCACCACTG
CTCCTGGGAGCTGGGGCCATCTATCTGTGGAGCCGCCAACAGCGTCGAAGG
GAGGCCAGAGGGGACTATAAGGACCATGATGGGGATTACAAGGATCACGA
CATTGACTACAAGGATGACGACGATAAAGGCGGTAGTGGCGGC

Table S1. Plasmids used in this study.

Plasmid code	Plasmid name	Promoter/terminator	Gene cloned	Referece
pYC1	pRS316-Mmm1	Own/Own	Mmm1	[34]
pYC1	pRS316-Mdm12	Own/Own	Mdm12	[34]
pYC91	pRS314-Vps13-D716H	Own/Own	Vps13-D716H	[7]
pYM21	pRS424-Mcp1	Own/Own	Mcp1	[14]
pYU41	pRS313-GPDp-MCS-CYC1ter	GPD/CYC1	None	This study
pYU47	pRS314-GPDp-MCS-CYC1ter	GPD/CYC1	None	This study
pYU53	pRS315-GPDp-MCS-CYC1ter	GPD/CYC1	None	This study
pYU54	pRS315-ADH1p-MCS-CYC1ter	ADH1/CYC1	None	This study
pYU59	pRS315-ADH1p-MCS-CYC1ter	GPD/CYC1	None	This study
pYU101	pFA6a-mScarlet-KanMX4	None/ADH1	mScarlet	This study
pSFL1	pTAC2-GFP1-10	None	GFP1-10	This study
pSFL2	pTAC2-GFP11	None	GFP11	This study
pSFL9	pRS316-GPDp-GFP1-10	GPD/CYC1	GFP1-10	This study
pSFL10	pRS314-GPDp-GFP1-10	GPD/CYC1	GFP1-10	This study
pSFL11	pRS316-GPDp-GFP11	GPD/CYC1	GFP11	This study
pSFL12	pRS314-GPDp-GFP11	GPD/CYC1	GFP11	This study
pSFL15	pRS314-GPDp-YNR021W-GFP11	GPD/CYC1	YNR021W-GFP11	This study
pSFL16	pRS316-GPDp-Ifa38-GFP1-10	GPD/CYC1	Ifa38-GFP1-10	This study
pSFL17	pRS315-GPDp-Ifa38-GFP1-10	GPD/CYC1	Ifa38-GFP1-10	This study
pSFL19	pRS316-GPDp-Vph1-GFP1-10	GPD/CYC1	Vph1-GFP1-10	This study
pSFL20	pRS315-GPDp-Vph1-GFP1-10	GPD/CYC1	Vph1-GFP1-10	This study
pSFL21	pRS314-GPDp-Vph1-GFP11	GPD/CYC1	Vph1-GFP11	This study
pSFL22	pRS316-GPDp-Tom71-GFP1-10	GPD/CYC1	Tom71-GFP1-10	This study
pSFL23	pRS314-GPDp-Tom71-GFP11	GPD/CYC1	Tom71-GFP11	This study
pSFL26	pRS316-GPDp-Pex3N-GFP1-10	GPD/CYC1	Pex3N-GFP1-10	This study
pSFL28	pRS316-GPDp-Erg6-GFP1-10	GPD/CYC1	Erg6-GFP1-10	This study
pSFL61	pRS316-GPDp-Ifa38-mCherry-GFP11	GPD/CYC1	IFA38-mCherry-GFP11	This study
pSFL66	pRS314-GPDp-Ifa38-3xFLAG-GFP11	GPD/CYC1	Ifa38-3xFLAG-GFP11	This study

pSFL67	pRS314-GPDp-Erg6-3xFLAG-GFP11	GPD/CYC1	Erg6-3xFLAG-GFP11	This study
pSFL69	pRS314-GPDp-Pex3N-3xFLAG-GFP11	GPD/CYC1	Pex3N-3xFLAG-GFP11	This study
pSFL73	pRS314-GPDp-3xFLAG-GFP11	GPD/CYC1	3xFLAG-GFP11	This study
pSFL74	pRS314-GPDp-V5-GFP11	GPD/CYC1	V5-GFP11	This study
pSFL75	pRS314-GPDp-Tom71-V5-GFP11	GPD/CYC1	Tom71-V5-GFP11	This study
pSFL84	pRS313-GPDp-Tom71-V5-GFP11	GPD/CYC1	Tom71-V5-GFP11	This study
pFL16	pRS315-GPDpBipN-mCherry-HDEL	GPD/CYC1	BipN-mCherry-HDEL	This study
pFL24	pRS315-ADH1p-mCherry-PTS1	ADH1/CYC1	mCherry-PTS1	This study
pFL72	pRS315-ADH1p-Erg6-mCherry	ADH1/CYC1	Erg6-mCherry	This study
pMM73	pCDNA3.1-Sec63(1-240)-V5-eGFP	CMV/bGH poly(A) signal	Sec63(1-240)-V5-eGFP	This study
pMM75	pCDNA3.1-Tom20(1-33)-FLAG-eGFP	CMV/bGH poly(A) signal	Tom20(1-33)-FLAG-eGFP	This study
pMM76	pCDNA3.1-Tom70(1-70)-FLAG-eGFP	CMV/bGH poly(A) signal	Tom70(1-70)-FLAG-eGFP	This study
pMM77	pCDNA3.1-ERdj1(1-200-)V5-eGFP	CMV/bGH poly(A) signal	ERj1(1-200-)V5-eGFP	This study
pMM80	pCDNA3.1_Tom20_GFP(1-10)	CMV/bGH poly(A) signal	Tom20(1-33)-FLAG-GFP1-10	This study
pMM82	pCDNA3.1_Tom70_GFP(1-10)	CMV/bGH poly(A) signal	Tom70(1-70)-FLAG-GFP1-10	This study
pMM87	pCDNA3.1_ERj1_GFP(11)	CMV/bGH poly(A) signal	ERj1(1-200-)V5-GFP11	This study
pMM89	pCDNA3.1_Sec63_GFP(11)	CMV/bGH poly(A) signal	Sec63(1-240)-V5-GFP11	This study

Table. S2. Primers used in this study.

Name	sequence (5'-3')
#YU291	AATTGCGGCCGCATGTCTAGTTCAATATTTGGCCC
#YU292	CCCGGATCCCTGAAATCTTGTTCTTTGCTTGTTTTTG
#YU293	AATTGCGGCCGCATGACTTTTATGCAACAGCTTC

#YU294	CCCGGATCCTTCCTTTTTAACCTGTCTTG
#YU295	AATTGCGGCCGCATGGCAGAGAAGGAGGAAGC
#YU296	CCCGGATCCGCTTGAAGCGGAAGAGCTTGC
#YU297	AATTGCGGCCGCATGGCCGAAAACTCCCTCCTG
#YU298	CCCGGATCCAAGCATGCCTTTAGCCCTATAAC
#YU305	AATTGCGGCCGCATGGCCCCAAATCAAAGATC
#YU306	CCCGGATCCGATCTGTTCTTTGATGAAGTG
#YU307	AATTGCGGCCGCATGAGTGAAACAGAATTGAG
#YU308	CCCGGATCCTTGAGTTGCTTCTTGGGAAG
#NU892	AAAGCGGCCGCATGTTTTTCAACAGACTAAG
#NU893	CCCACTAGTTTCTACATCATCGGCACCTCT
#NU946	CCCACTAGTATGGTGAGCAAGGGCGAGGAG
#NU948	AAACTCGAGTTACAATTCATCATGCTTGTACAGCTCGTCCATGCC
#YU377	CCCGGATCCATGGTGAGCAAGGGCGAGGAGGAT
#YU378	CCCGAATTCTTACAATTTTGAGCCACCAGACCCTCCCTTGTACAGCTCGTCCATGCC
#NU539	CCCACTAGTATGGTGAGCAAGGGCGAGGAGGATAAC
#NU540	CCCCGGATCCTTGTACAGCTCGTCCATGCCGCCGG
#YU1006	CATAGGATCCTTACTTTTCGTTGGGATCTTTCGAA
#YU1007	CATAGGATCCTTATGTAATCCCAGCAGCATTTACG
#YU1008	CCCTCTAGAGCCACCATGGGTGGCACTAG
#YU1009	CCCTCTAGAGCCACCATGGATGGAGGGTC
